# 

**Published:** 1903-08-29

**Authors:** 


### NOTICE TO CORRESPONDENTS.

All MSS., Letters, Books for Review, and other matters intended for the Editor should be addressed The Editor, "The Hospital," The Hospital Buildings, 28 & 29 Southampton Street, Strand, W.C.

The Editor cannot undertake to return rejected MS., even when accompanied by stamped directed envelope.

**Notice to Advertisers and Subscribers.**

All Advertisements, Orders for Copies of The Hospital, and Business Communications should be addressed to **THE MANAGER, (Not to the Editor)**, The Hospital, 28 & 29 Southampton Street, Strand, London, W.C.

The Cost of Subscription, post free, is as follows.—Per week, 3½d.; per quarter, 3s. 9d.; per half-year, 7s. 6d.; per annum, 15s. Foreign and Colonial:—Per annum, 19s. 6d., payable, in all cases, in advance.

**NOTICE.—Vol. XXXIII. of "The Hospital" (October 1902 to March 1903) in handsome cloth binding, will shortly be on sale at the office of this Journal, price 7s. 6d. Cases for binding the Numbers contained in this Volume 1s. 6d. Index to Vol. XXXIII., 2½d., post free.**

**The monthly part for August is now ready. Price 1s., by post 1s. 3d.**

### THE STUDENT OF MEDICINE.

### APPOINTMENTS.

**Henry MacCormac**, M.B., Ch.B.Edin., House Physician to the City of London Hospital for Diseases of the Chest, Victoria Park, E. **Hugh Lennox Munro**, M.B., Ch.B.Edin., House Surgeon, Royal Infirmary, Lancaster. **Llewellyn Phillips**, M.D., B.C., M.R.C.P., F.R.C.S., Professor of Clinical Medicine in the School of Medicine at Cairo, and Second Physician to Kasr el Ainy Hospital. **Joseph Henry Thompson**, M.B., Ch., B.A.O., R.U.I., Junior Assistant House Surgeon to the Stockport Infirmary.

### "The Hospital" Institutional Library.

"Hospitals and Asylums of the World." 4 Vols., with a Portfolio of Plans, complete £12 12s.

"Burdett's Hospitals and Charities, 1903." 5s. net.

"Burdett's Official Nursing Directory, 1903." 3s. net.

"Cottage Hospitals, General, Fever, and Convalescent." 10s. 6d.

"Uniform System of Accounts for Hospitals and Public Institutions." New Edition. 4s. net.

"Hospital Expenditure: The Commissariat." 2s. 6d.

"A Legal Handbook for the Use of Hospital Authorities." 2s. 6d.

"The Cottage Hospital Case Book or Register of Patients." 10s. and 12s. 6d.

All these are published by the SCIENTIFIC PRESS, Ltd., and may be obtained through any bookseller, or direct from the publishers, 28 and 29 Southampton Street, Strand, London, W.C.

THE SCIENTIFIC PRESS, LIMITED,
28 & 29 Southampton Street, Strand, London, W.C.

### Special Text-Books for Nurses.

### OPHTHALMIC NURSING.

By SYDNEY STEPHENSON, F.R.C.S.E.

Price 3/6 net, 3/10 post free.

A valuable guide on the Nursing of Eye Cases.

### NURSING IN DISEASES OF THE THROAT, NOSE, AND EAR.

By P. MACLEOD YEARSLEY, F.R.C.S.

Price 2/6.

Contains useful hints on these special subjects.

### THE ART OF MASSAGE.

By A. CREIGHTON HALE.

Price 6/-.

Every Nurse must find a knowledge of Massage useful and interesting.

### FEVERS AND INFECTIOUS DISEASES.

By Dr. WILLIAM HARDING.

Price 1/-.

An excellent little handbook.

### NOTES ON PHARMACY AND DISPENSING.

By C. J. S. THOMPSON.

Price 1/-.

An admirable introduction to Dispensing.

### MENTAL NURSING.

By Dr. WILLIAM HARDING.

Price 1/-.

Full of useful advice to Nurses in Mental Cases.

### OUTLINES OF INSANITY.

By Dr. WALMSLEY.

A Treatise on the Salient Features of Insanity. Very useful to the intelligent Mental Nurse.

*Obtainable of all Booksellers, and published by*
THE SCIENTIFIC PRESS, LIMITED, 28 & 29 Southampton Street, Strand, London,
*who will send their latest Catalogue of Nursing Text-books post free to any Nurse on application.*

